# Case report of the duplex vermiform appendix: A rare presentation in an elderly patient

**DOI:** 10.1016/j.ijscr.2022.106829

**Published:** 2022-02-12

**Authors:** Amenah Dhannoon, Mobarak Kunna, Dawn Baynes, Seán O. Hynes, Emmeline Nugent

**Affiliations:** a Royal College of Surgeons in Ireland, 123 St Stephen's Green, Dublin, Ireland; b Department of Surgery, University Hospital Galway, Galway, Ireland; c Department of Histopathology, Division of Anatomic Pathology, University Hospital Galway, Galway, Ireland; d Discipline of Pathology, School of Medicine, National University of Ireland Galway, Galway, Ireland; e Academic Department of Surgery, National University of Ireland Galway, Galway, Ireland

**Keywords:** Duplex appendix, Duplicated appendix, Appendiceal mass, Case report

## Abstract

**Introduction and importance:**

Duplex appendix is a rare anatomical entity with incidence rate of 0.004 and 0.009%. Diagnosis is often missed despite growth in radiological investigations. Missed appendiceal anomalies can lead to undesirable medicolegal implications.

**Case presentation:**

Here we discuss a case of a 76-year-old-male who initially presented to his primary care physician with right-sided abdominal pain for several weeks. A colonoscopy was performed and demonstrated a lesion arising from the appendicular orifice. The patient underwent staging imaging including Computerised Tomography of the abdomen and pelvis which demonstrated a dilated appendix. The patient underwent a laparoscopic right hemicolectomy. He made an uneventful recovery post-operatively and at his follow-up review at 4 weeks and 2 months.

**Discussion:**

While duplex appendix has been reported in the literature, to our knowledge this is the first case report to describe duplicated appendix presenting as a colonic mass in an elderly patient.

**Conclusion:**

Intra-operative examination of the cecum is paramount to rule out appendiceal anomalies and prevent medicolegal complications.

## Highlights

• Duplex appendix is a rare entity with incidence rate of 0.004–0.009%.
• Diagnosis of duplicated appendix is often missed despite growth in radiological investigation.
• Missed appendiceal anomalies can lead to undesirable medicolegal implications.
• Intra-operative examination is the only reliable option to rule out appendiceal anomalies.

## 1 Introduction

Duplicated appendix is a very rare anatomical entity with significant clinical and surgical relevance. It was first described in foetal studies in 1867 by Bartles and in adult studies in 1892 by Picoli [1]. The reported incidence rate is 0.004 and 0.009% [2], [3].

Several classifications have been described to classify a double appendix. The most commonly used is the Cave–Wallbridge classification [4]. These classification are A, B1, B2 and C. However this has been updated to include other subtypes like the horseshoe appendix and triplet appendix [4], [5] (see Table 1). The diagnosis can either be an incidental finding on imaging or through complicated presentations. Among these complications are acute appendicitis, colonic perforation, obstruction, intussusception, bleeding, pain, failure to thrive and abdominal mass [4], [5], [6], [7].

**Table 1:**
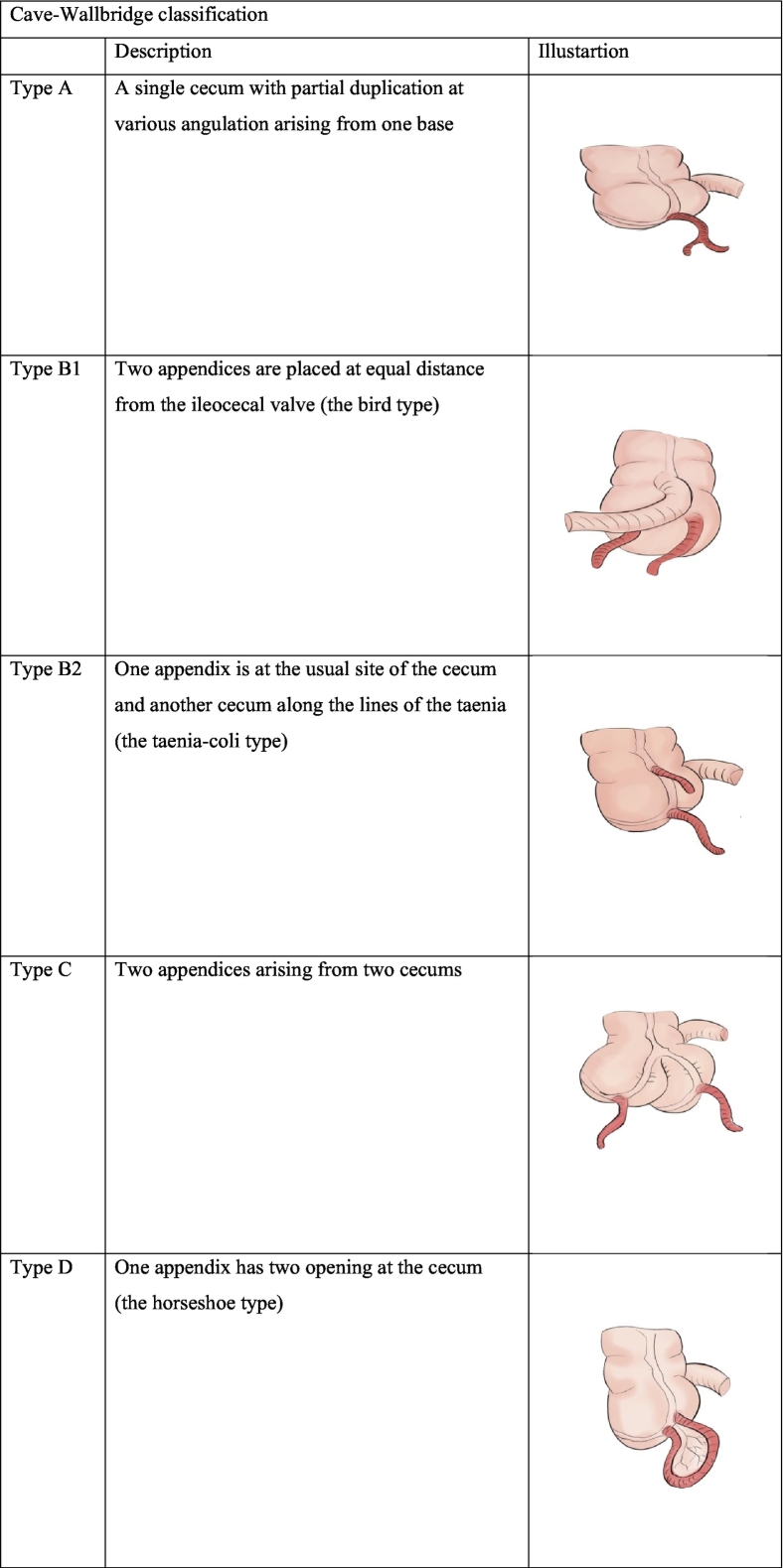
Showing the modified Cave-Wallbridge classification [4], [5].

Despite the rarity of this presentation, knowledge of this anatomical entity is of paramount importance to prevent complications that could arise if the duplex appendix was unrecognised and the medico-legal implication associated with this. We present a case of the eldest patient reported to present with this anomaly.

## 2 Case report

A 76-year-old-male presented to his General Practitioner (GP) complaining of right-sided abdominal pain that had been ongoing for more than 4 weeks. His medical background was significant for hypercholesteraemia and a long history of pipe-smoking. His medications are limited to oral atorvastatin 20 mg once daily. He was referred to the endoscopy unit to undergo colonoscopy. During the procedure, a polypoid lesion was noted to be protruding from the appendiceal orifice. There was clinical concern that it represented an adenomatous polyp or malignancy therefore biopsies were taken. Due to its location, it was not feasible to endoscopically remove the lesion. Biopsies demonstrated benign colonic tissue only. A Computed Tomography (CT) of the abdomen and pelvis demonstrated dilatation of the appendiceal orifice of more than 2 cm, a thickened irregular appearing appendix and an irregular soft tissue nodule measuring 1.2 cm adjacent to the tip and abutting the bladder. His laboratory tests were within the normal range. The case was discussed at the weekly multi-disciplinary team meeting. Given the unusual overall endoscopic and imaging findings where the concern was for a malignant process, in addition to the patient's symptoms the decision was made to perform a laparoscopic right hemicolectomy by a consultant colorectal surgeon. At surgery the appendix mass was densely adherent to the bladder peritoneum and was carefully dissected free.

**Fig. 1:**
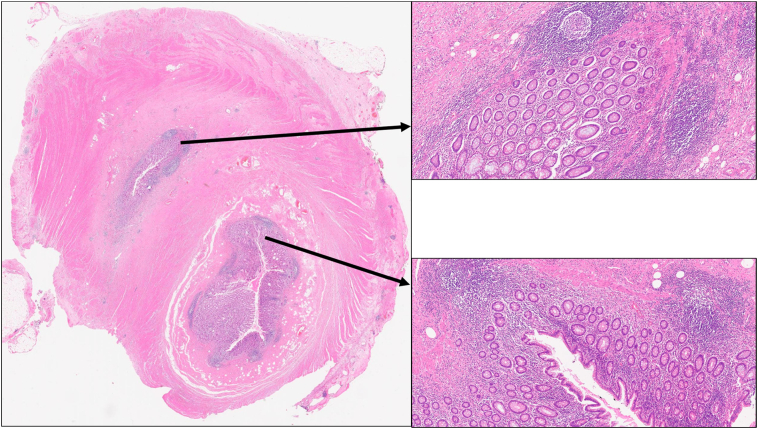
H&E whole slide image at 1× demonstrating the presence of a double lumen in the appendix in its upper third, the insets show the presence of normal appendiceal tissue on higher power (10×).

**Fig. 2:**
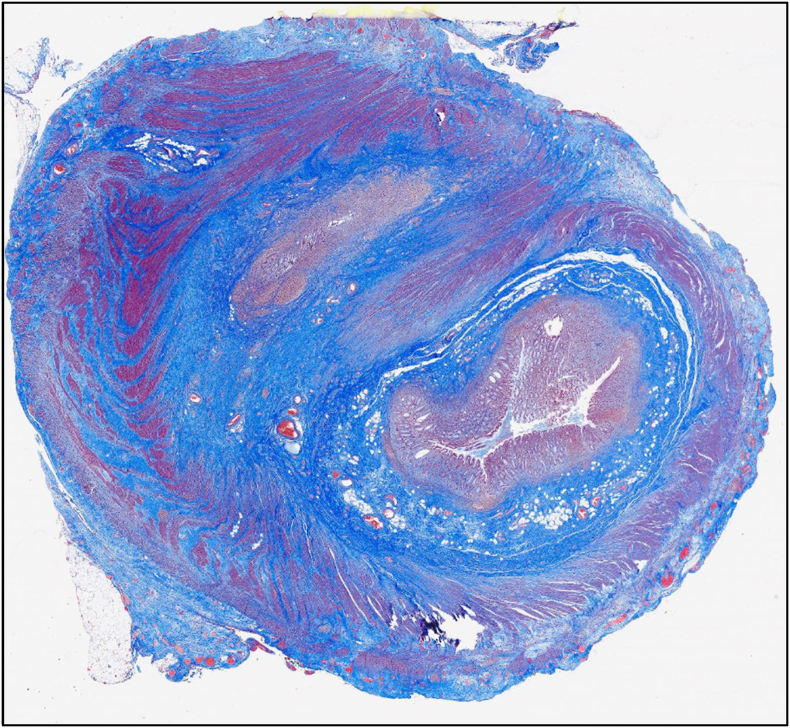
Whole slide scan at 1× showing Mason's trichrome staining of the double lumen demonstrating the presence of a complete wall for both lumens.

The microscopic examination confirmed an overall double lumen in the appendix, furthermore the separate nodule that was attached the tip of the appendix represented a further nodule of appendiceal tissue (Fig. 1, Fig. 2). Special stains showed a thickened fibrotic wall but there was no infiltration of the wall by a pathological process, e.g. amyloid. No mass was identified to suggest an intussusception of the appendix. The histological features confirmed two separate lumens. Moreover, the separate nodule of appendiceal tissue was thought to represent autoamputation of the tip but no faecal material was present to suggest it was previously intact with the main appendix. It may represent a developmental anomaly.

The patient made an uneventful recovery and was discharged home on day ten and was able to return to his normal living activities at his 4-week and 2-month clinic review.

## 3 Discussion

The vermiform appendix is a caecal diverticulum of variable sites and embryological anomalies. Duplication of the appendix is one of the rare congenital variables. According to Nageswaran et al., there were 141 cases have been reported in the literature with variable presentations and mostly diagnosed intra-operatively with male: female ratio of 1.4:1. While 80% of gastrointestinal system duplication present before the age of two years [7], the reported age for duplicated appendix ranges between foetus and 69 [8]. The most commonly reported Cave–Wallbridge category was type A [8]. Our case is the first case to report this anomaly in a person older than 69 presented with abdominal pain and a colonic mass on endoscopic examination.

Our case is unusual in that it is not clear why the patient developed symptoms from this congenital anomaly in his 60's. The pathological findings suggest that the duplex appendix had autoamputated and this could account for the clinical symptom of abdominal pain. Intussusception as a cause of abdominal pain remains a possibility also however no lead point was identified either radiologically or pathologically.

Awareness of this rare anomaly is of paramount importance to consider in a patient with previous appendicectomy if they present with clinical symptoms and signs of appendicitis [9]. Similarly, it is also important to consider this entity in elderly patients presenting with a colonic mass or abdominal pain.

In conclusion, our case highlights that duplicated appendix may present in elderly people who would have had lived decades without any consequences from this anomaly. Furthermore, despite the growing dependency on radiological investigation to diagnose appendiceal pathologies, diagnosis of duplicated appendix is often missed [7], therefore thorough intra-operative examination of the caecum is the only reliable option to rule out appendiceal anomalies and prevent medicolegal complications.

The case has been reported in line with the SCARE 2020 criteria [10].

## Sources of funding

None.

## Ethical approval

None.

## Consent

Written informed consent was obtained from the patient for publication of this case report and accompanying images. A copy of the written consent is available for review by the Editor-in-Chief of this journal on request.

## Research registration

None.

## Guarantor

Amenah Dhannoon.

## Provenance and peer review

Not commissioned, externally peer-reviewed.

## Patient consent

Patient written consent has been obtained to publish this case.

## CRediT authorship contribution statement

Amenah Dhannoon: Writing- Original draft preparation, Visualisation.

Mubarak Kunna: Conceptualization.

Dawn Bynes: Resources.

Séan Hynes: Reviewing and Editing.

Emmeline Nugent: Operating surgeon, Supervision.

## Declaration of competing interest

None.
